# Archigregarines of the English Channel revisited: New molecular data on *Selenidium* species including early described and new species and the uncertainties of phylogenetic relationships

**DOI:** 10.1371/journal.pone.0187430

**Published:** 2017-11-03

**Authors:** Sonja Rueckert, Aleš Horák

**Affiliations:** 1 School of Applied Sciences, Edinburgh Napier University, Edinburgh, United Kingdom; 2 Biology Centre of CAS, Institute of Parasitology, České Budějovice, Czech Republic; 3 University of South Bohemia, Faculty of Science, České Budějovice, Czech Republic; University of Cambridge, UNITED KINGDOM

## Abstract

**Background:**

Gregarines represent an important transition step from free-living predatory (colpodellids *s*.*l*.) and/or photosynthetic (*Chromera* and *Vitrella*) apicomplexan lineages to the most important pathogens, obligate intracellular parasites of humans and domestic animals such as coccidians and haemosporidians (*Plasmodium*, *Toxoplasma*, *Eimeria*, *Babesia*, etc.). While dozens of genomes of other apicomplexan groups are available, gregarines are barely entering the molecular age. Among the gregarines, archigregarines possess a unique mixture of ancestral (myzocytosis) and derived (lack of apicoplast, presence of subpellicular microtubules) features.

**Methodology/Principal findings:**

In this study we revisited five of the early-described species of the genus *Selenidium* including the type species *Selenidium pendula*, with special focus on surface ultrastructure and molecular data. We were also able to describe three new species within this genus. All species were characterized at morphological (light and scanning electron microscopy data) and molecular (SSU rDNA sequence data) levels. Gregarine specimens were isolated from polychaete hosts collected from the English Channel near the Station Biologique de Roscoff, France: *Selenidium pendula* from *Scolelepis squamata*, *S*. *hollandei* and *S*. *sabellariae* from *Sabellaria alveolata*, *S*. *sabellae* from *Sabella pavonina*, *Selenidium fallax* from *Cirriformia tentaculata*, *S*. *spiralis* sp. n. and *S*. *antevariabilis* sp. n. from *Amphitritides gracilis*, and *S*. *opheliae* sp. n. from *Ophelia roscoffensis*. Molecular phylogenetic analyses of these data showed archigregarines clustering into five separate clades and support previous doubts about their monophyly.

**Conclusions/Significance:**

Our phylogenies using the extended gregarine sampling show that the archigregarines are indeed not monophyletic with one strongly supported clade of *Selenidium* sequences around the type species *S*. *pendula*. We suggest the revision of the whole archigregarine taxonomy with only the species within this clade remaining in the genus *Selenidium*, while the other species should be moved into newly erected genera. However, the SSU rDNA phylogenies show very clearly that the tree topology and therefore the inferred relationships within and in between clades are unstable and such revision would be problematic without additional sequence data.

## Introduction

Gregarine apicomplexans are obligate, unicellular parasites of freshwater, marine and terrestrial invertebrates infecting the intestine, coelom and reproductive vesicles. As gregarines do not generally cause any harmful diseases compared to their well-studied closely related sistergroups known as notorious parasites of humans and domestic animals (e.g. *Plasmodium*, *Cryptosporidium* and *Toxoplasma*), they are still an undersampled and little described group of protists. There is a strong need to overhaul the complete gregarine taxonomy and Cavalier-Smith [1] made an attempt in doing so. Unfortunately, his way to tackle the problem by erecting 18 new taxa mainly based on a single SSU rDNA analysis is rather preliminary. The poor support of most of the clades and branching orders in question does not allow for these taxonomic conclusions to be made. At this point, we will therefore stick with the separation of the gregarines into the three major groups (archigregarines, eugregarines and neogregarines) based on trophozoite morphology, host affinity and life history (e.g. [2–9])

The first time a gregarine was officially described was in 1828 by Dufour, who erected the genus *Gregarina* [2]. While in the early years of gregarine discovery, morphological descriptions were based exclusively on light microscopic and histologic studies, the importance of ultrastructural studies using electron microscopy became quickly apparent and shed important light on differences e.g. between archigregarines and eugregarines (e.g. [10–15]). These data showed that archigregarines have wide, longitudinal epicytic folds with an underlying layer of subpellicular microtubules, while eugregarines have narrow epicytic folds with apical filaments and rippled dense structures (see [16]). Nonetheless, available data are still quite limited. In the last decades the molecular approach for phylogenetic analyses started to boom and several genomes of the crown apicomplexans including *Plasmodium*, *Toxoplasma*, *Crytosporidium*, *Babesia*, etc. are already available (e.g. [17,18]). The genomes of photosynthetic apicomplexan lineages including *Chromera velia* and *Vitrella brassicaformis* have been published recently [19], but gregarine apicomplexans have hardly entered the genomic age (apart from a very partial genome draft of *Ascogregarina taiwanensis* [20]). The importance of molecular phylogenetic data and their value in inferring relationships between gregarine species has lead to an increase in available sequence data in public databases such as GenBank, but these are still very limited in numbers and mostly restricted to SSU rDNA sequences. One general problem with molecular phylogenies is that the outcome often heavily depends on the original species dataset utilized for the analyses [21].

Archigregarines, which are found only in marine invertebrates, especially polychaetes, are of particular interest, as they are supposed to form the paraphyletic stem group from which all other gregarines and maybe even all apicomplexans have evolved [2,22–26]. Archigregarines possess a unique mixture of ancestral (myzocytosis; e.g. [27]) and derived (lack of apicoplast, presence of subpellicular microtubules; e.g. [3, 28,29]) features. There are around ~80 archigregarine species described to date [11,25,26,29]. Around 65 described species belong to the genus *Selenidium*. Levine [3] proposed to erect a new genus *Selenidioides* to accommodate those 11 species that show merogony, belonging truly to the archigregarines, while the rest of the *Selenidium* species was shuffled into the order Eugregarinorida. Because it is difficult to prove or disprove the presence or absence of merogony/schizogony (asexual reproduction of trophozoite stage) within a lifecycle, we and many other authors do not believe it to be a good indicator for the relationships among archigregarines [16,25,26,29–32]. Therefore, we will not follow this split of *Selenidium* species and thus treat *Selenidium* as the main genus of archigregarines. Just recently Schrével et al. [33] published for the first time molecular data for the type species *Selenidium pendula*. The presented phylogenies show one clade of true Selenidiidae around *S*. *pendula* and two other clades of archigregarines, all reflecting their respective host species.

In this study we set out to obtain molecular data of morphologically and ultrastructurally well-described species including the type species *Selenidium pendula*, *S*. *hollandei*, *S*. *sabellariae*, *S*. *sabellae* and *S*. *fallax*. In addition, we were able to discover three new species. We will present morphological characterizations of all species by light and scanning electron microscopy and discuss the phylogenetic relationships of archigregarines within the context of a broad apicomplexan phylogeny based on SSU rDNA data.

## Material and methods

### Collection and isolation of organisms

Different polychaete species were collected by hand from rocks and sandy beaches from the English Channel near the Station Biologique Roscoff (SBR), France, in May 2013 and in June 2014. No specific permits were required for the sampling locations as they are not privately owned or protected. This study did not involve any endangered or protected species. The collected polychaetes were identified based on morphological characteristics. *Selenidium pendula* was isolated from the intestines of *Scolelepis squamata* (Mueller, 1806) (Spionida) collected by hand from a beach close to the SBR (48°43´31´´N, 3°59´26´´W). *Selenidium hollandei* and *S*. *sabellariae* were isolated from the intestines of *Sabellaria alveolata* (Linnaeus, 1767) (Sabellida) collected by hand from Saint Efflam (48°68´48´´N, 3°61´13´´W). *Selenidium sabellae* was isolated from the intestines of *Sabella pavonina* Savigny, 1822 (also Sabellida) hand-collected from Pempoul (48°68´23´´N, 3°95´14´´W). *Selenidium fallax* was isolated from *Cirriformia tentaculata* (Montagu, 1808), *S*. *spiralis* sp. n. and *S*. *antevariabilis* sp. n. were isolated from *Amphitritides gracilis* (Grube, 1860) (both Terebellida) both polychaete species were collected by hand from rock assemblages close to the SBR (48°43’44”N, 3°59’23”W). *Selenidium opheliae* sp. n. was isolated from *Ophelia roscoffensis* Augener, 1910 (Opheliidae) hand-collected from Le Guillec (48°68´56´´N, 4°06´78´´W).

The trophozoites of each species were released in seawater by teasing apart the intestines of the respective host with fine-tipped forceps under a dissecting microscope (Zeiss Stemi 2000). The gut material was examined under an inverted microscope (Zeiss Axiovert A1) and parasites were isolated with a hand drawn glass pipette and washed three times in filtered seawater, before being examined and photographed under the inverted microscope or prepared for DNA extraction.

### Light and scanning electron microscopy

Differential interference contrast (DIC) and phase contrast (PC) light micrographs of all species were taken with a 5 megapixel CMOS camera AxioCam ERc 5s, attached to an inverted microscope (Zeiss Axiovert 1).

Between 8 and 60 specimens each of the isolated gregarine species were prepared for scanning electron microscopy (SEM). Some individuals were deposited directly into the threaded hole of separate Swinnex filter holders, containing a 10 μm polycarbonate membrane filter (Millipore Corp., Billerica, MA), that was submerged in 10 ml of seawater within a small canister (2 cm diam. and 3.5 cm tall). A piece of Whatman No. 1 filter paper was mounted on the inside base of a beaker (4 cm diam. and 5 cm tall) that was slightly larger than the canister. The Whatman filter paper was saturated with 4% (w/v) OsO_4_ and the beaker was turned over the canister. The parasites were fixed by OsO_4_ vapors for 30 min. Ten drops of 4% (w/v) OsO_4_ were added directly to the seawater and the parasites were fixed for an additional 30 min. A 10-ml syringe filled with distilled water was screwed to the Swinnex filter holder and the entire apparatus was removed from the canister containing seawater and fixative. Other individuals were deposited in hand-made baskets (top end of a 1000μl pipette tip fixed with silicon to the same 10 μm filters) and placed in 24-well culture plates. These filters were prefixed in 2.5% (v/v) glutaraldehyde in 0.05 M cacodylate buffer (pH = 7.4) for one hour, washed and post-fixed with 1% (w/v) OsO_4_ for 45 minutes. All filters were washed with water and dehydrated with a graded series of ethyl alcohol. Filters prepared in the Swinnex filter holders were critical point dried with CO_2_, filters from the hand-made baskets were air-dried. Filters were mounted on stubs, sputter coated with 5 nm of gold, and viewed under a scanning electron microscope (JEOL JBM7401F or Phenom 806). Some SEM data were presented on a black background using Adobe Photoshop CS5 (Adobe Systems Incorporated, San Jose, CA).

### DNA isolation, PCR, cloning, and sequencing

Individual trophozoites of each species were isolated from the dissected hosts, washed three times in filtered seawater, and deposited into a 1.5-ml microcentrifuge tube: 23 trophozoites of *S*. *pendula* from *S*. *squamata*, 27 trophozoites of *S*. *hollandei* and 75 trophozoites of *S*. *sabellariae* from *Sabellaria alveolata*, 8 trophozoites of *S*. *sabellae* from *Sabella pavonina*, 45 cells of *Selenidium fallax* from *Cirriformia tentaculata*, 11 and 12 trophozoites of *S*. *spiralis* sp. n. and *S*. *antevariabilis* sp. n. from *Amphitritides gracilis*, and 31 trophozoites of *S*. *opheliae* sp. n. from *Ophelia* sp.

DNA was extracted using the MasterPure^TM^ Complete DNA and RNA Purification Kit (Epicentre Biotechnologies, Madison, WI). Small subunit rDNA (SSU rDNA) sequences were PCR-amplified either using a total volume of 50 μl containing 1 μl of primer, 5 μl of DNA template, 25 μl of OneTaq Mastermix (New England Biolabs, Inc., Ipswich, USA), or using a total volume of 25μl containing 2 μl of primer, 2.5 μl of DNA template, 20.5 μl of dH_2_O and one PuReTaq Ready-to-go PCR Bead (GE Healthcare, Quebec, Canada).

The SSU rDNA sequences from these species were amplified in one fragment (~1800 bp) using universal eukaryotic PCR primers F1 5´-GCGCTACCTGGTTGATCCTGCC-3´ and R1 5´-GATCCTTCTGCAGGTTCACCTAC-3´ [34] and internal primers designed to match existing eukaryotic SSU sequences F2 5´-AAGTCTGGTGCCAGCAGCC-3´ and R2 5´-TTTAAGTTTCAGCCTTGCG-3´. PCR was performed using MJ Mini^TM^ Gradient Thermal Cycler (Bio-Rad) and the following protocol: After 4 cycles of initial denaturation at 94 ^o^C for 4.5 min, 45 ^o^C for 1 min and 72 ^o^C for 1.75 min, 34 cycles of 94 ^o^C for 30 sec (denaturation), 50 ^o^C for 1 min (annealing), 72 ^o^C for 1.75 min (extension), followed by a final extension period at 72 ^o^C for 10 min. PCR products corresponding to the expected size were gel isolated using the UltraClean^TM^ 15 DNA Purification kit (MO Bio, Carlsbad, California) and cloned into the pCR 2.1 vector using the TOPO TA cloning kit (Invitrogen, Frederick, MD). Eight cloned plasmids were digested with *Eco*RI and screened for size. One or two clones for each species were sequenced with ABI big dye reaction mix using vector primers and internal primers oriented in both directions.

The new SSU rDNA sequences were initially identified by BLAST analysis and subsequently verified with molecular phylogenetic analyses (GenBank Accession numbers: *Selenidium pendula* MF882901, *Selenidium hollandei* MF882899, *Selenidium sabellariae* MF882900, *Selenidium sabellae* MF882906, *Selenidium fallax* MF882905, *Selenidium spiralis sp*. *n*. MF882902, *Selenidium antevariabilis* sp. n. MF882903, *Selenidium opheliae* sp. n. MF882904).

### Molecular phylogenetic analysis

The eight new SSU rDNA sequences were aligned with 163 other SSU rDNA sequences, representing the major lineages of apicomplexans (with an emphasis on gregarines) and relevant outgroups (ciliates, dinozoans and chromerids and colpodellids), using local alignment with generalized affine gap costs (E-INS-I) as implemented in MAFFT [35]. Sites comprised mostly of gaps and ambiguously aligned regions were manually excluded from the 171-taxon alignment in SEAVIEW 4 [36] resulting in 1,488 unambiguously aligned sites (a NEXUS file of this alignment is available upon request). Alternatively, we have used trimAl 1.2 [37] for automatic ambiguous sites detection/exclusion under relaxed (parameters set as '-gt 0.3' and '-st 0.001'; dataset R, 1805 sites) as well as strict (-gappyout option engaged; dataset G, 945 sites) settings. Based on the results of a preliminary analyses and in agreement with previously published studies, we have also created a smaller dataset (S) based on the exhaustive sampling of Selenidiidae s.s. (as defined by [33]), as well as other archigregarine taxa (81 taxa, 1601 sites) rooted with marine eugregarines. Homogeneity of base-composition was tested using Tree-Puzzle 5.3 [38]. Root-to-tip distances for main clades of the dataset were measured using the TreeStat 1.2 (http://tree.bio.ed.ac.uk/software/treestat/).

Because the 18S rDNA of several gregarine taxa did not pass the homogeneity test, the Bayesian inference was carried out with exchange rates defined by the general time-reversible model and the number of categories limited to 40 (GTR + C40), implemented in Phylobayes 4.1 [39]. This model was chosen as a compromise between the robustness of CAT admixture model with an infinite number of rate categories, which is however mostly suitable for large phylogenomic datasets due to its complexity, and simplicity of time-tested GTR model. Two chains were run until they converged (i.e. the maximum observed discrepancy below 0.2 and effective number of model parameters reached 100). Posterior probabilities of the branching were reconstructed after burn-in of the first fifth of the generations. Alternatively, we have also performed a maximum likelihood analysis under the gamma-corrected GTR model using the RAxML 8.2a [40]. The highest-scoring topology was estimated using the rapid-bootstrapping algorithm from 1000 replicates, the branching support was assessed using the non-parametric bootstrapping and the ‘thorough’ algorithm from 1000 replicates using the same model and software. The putative monophyly of archigregarines was tested using the approximate likelihood ratio test as implemented in Consel [41]. Prior to the analysis, we have optimized the topology and branch lengths of the starting tree with forced monophyly of archigregarine sequences in RAxML.

### Nomenclatural acts

The electronic edition of this article conforms to the requirements of the amended International Code of Zoological Nomenclature, and hence the new names contained herein are available under that Code from the electronic edition of this article. This published work and the nomenclatural acts it contains have been registered in ZooBank, the online registration system for the ICZN. The ZooBank LSIDs (Life Science Identifiers) can be resolved and the associated information viewed through any standard web browser by appending the LSID to the prefix "http://zoobank.org/". The LSID for this publication is: urn:lsid:zoobank.org:pub:91B0E976-C459-46E5-9B93-FA96177BC9CA. The electronic edition of this work was published in a journal with an ISSN, and has been archived and is available from the following digital repositories: PubMed Central, Edinburgh Napier Repository (http://www.napier.ac.uk/research-and-innovation/repository).

## Results

### Morphological observations

All given measurements are based on light micrographs from fresh material, as some of the gregarines seemed to have shrunk during preparation for scanning electron microscopy. All trophozoites were brownish in colour under the LM, reflecting an accumulation of amylopectin granules within the cytoplasm.

**Fig 1:**
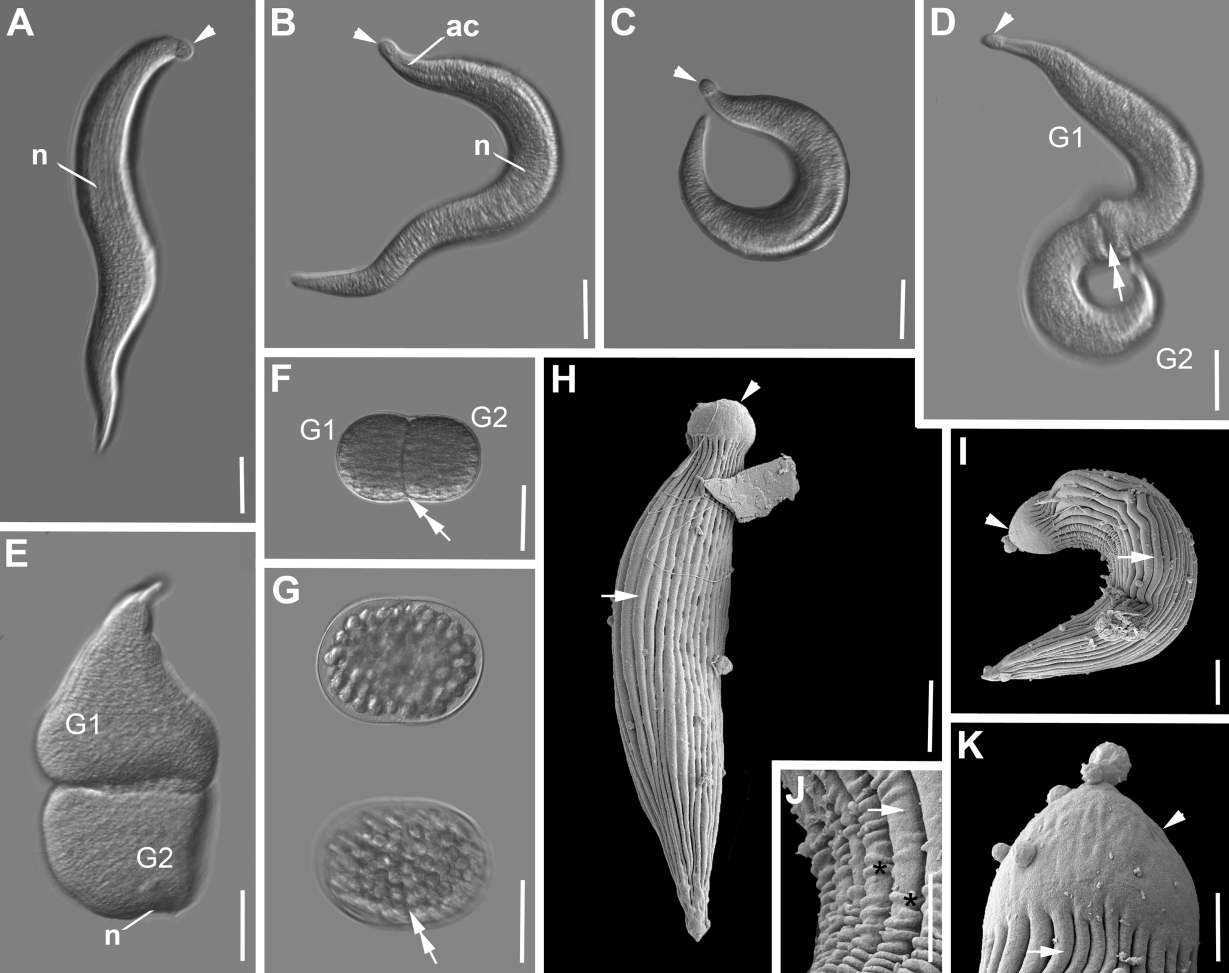
Differential interference contrast (DIC) light micrographs and scanning electron micrographs (SEM) showing the general morphology and surface ultrastructure of the gregarine *Selenidium pendula* isolated from the polychaete *Scolelepis squamata*. **A-C.** DIC micrographs showing a spindle-shaped trophozoite in different positions of the pendular movement. The mucron at the anterior end is rounded (arrowhead) and visually separated from the rest of the cell with a slight restriction. The axial canal (ac) is conspicuous at the anterior end, but ran along the entire cell. The oval nucleus (n) is situated in the middle of the cell. **D-E.** Two gamonts (G1, G2) in caudal syzygy. The mucrons (arrowhead) are visible. The double arrow marks the junction between the two gamonts. Gamonts are changing from elongated to stumpy. **F.** Young gametocyst, the junction (double arrow) is still visible. **G.** Gametocysts in different focal planes, packed with round oocysts. **H-K.** SEM micrographs of trophozoites showing the epicytic longitudinal folds (arrow), the mucron (arrowhead) free of folds and some transverse striations in the inner curvature of the cell. Scale bars: Fig 1A-E, 30 μm; Fig 1F, 50 μm; Fig 1G, 70 μm; Fig 1H-I, 5μm; Fig J-K, 2μm.

***Selenidium pendula*** (Fig 1, Table 1). Trophozoites were isolated from the polychaete *Scolelepis squamata* (Mueller, 1806). The morphology matched the original description of *S*. *pendula* by Giard [42] and several accounts in studies on different aspects that followed [10,11,33,43]). The cells were elongated and spindle-shaped (Fig 1). Trophozoites were 200 μm (147–260 μm, n = 15) long and 25 μm (12–48 μm, n = 15) wide. There was a slight restriction between the anterior end and the rest of the body. The anterior end was globular and rounded, while the posterior end was tapering into a pointed tip (Fig 1A–1C, 1H and 1I). The axial canal was visible along the entire cell starting from the restriction at the anterior end to the tip of the posterior end (Fig 1B). The oval nucleus [20 (16–28) μm x 13 (7–22), n = 10] was situated in the middle of the cell (Fig 1A and 1B). Mature trophozoites (or gamonts) pair up in caudal syzygy (Fig 1D). The two individuals in syzygy underwent continuous change from elongated cells, which showed active movement to more stumpy cells (Fig 1E), which did show restricted movement. Different developmental stages of the gamontocyst and gametocyst were observed (Fig 1F and 1G). The gametocyst was oval [129 (103–146) μm x 88 (61–106) μm, n = 5]. The junction between the two cells was still visible in the young gametocyst. (Fig 1F). At the end of sporogony the gametocyst was packed with spherical oocysts (Fig 1G). The oocysts containing 4 sporozoites were 13.1 μm in diameter (11.8–14.5μm, n = 22). The SEM micrographs demonstrated that there were around 30–34 longitudinal epicytic folds inscribing the surface of the trophozoites. The anterior end was free of folds (Fig 1H, 1I and 1K), while some of the folds terminated towards the posterior end. In bended and shortened cells, transverse folds appeared on the longitudinal epicytic folds (Fig 1K). In the middle of the cell, the density of longitudinal folds was 1 fold/micron (Fig 1J). Single trophozoites and two individuals in early syzygy were capable of pendular movement, which gave this type species of *Selendium* its name *S*. *pendula* [42].

**Table 1:** Morphological comparison of *Selenidium* species presented in this study and other species that belong to the same clades in the phylogenetic tree (data from this study highlighted in bold).

*species*	*Host*	Locality	Host tissue	Trophozoite shape	Trophozoite size (L x W, μm)	Nucleus shape	Nucleus size (L x W, μm)	Position of nucleus	Motility	# of long. epicytic folds	Trans. surface folds	Shape of mucron	SSU rDNA	Ref.
*S. pendula*	*Nerine cirratulus*	E. Atlantic	Intestine	spindle-shaped	180 x 30–40	round to oval	18–33 x 13–32	middle	bending, twisting, pendulum-like	20–30	?	pointed	-	[3,43]
***S. pendula***	***Scolelepis squamata***	**E. Atlantic**	**Intestine**	**spindle-shaped**	**147–260 x 12–48**	**oval**	**16–28 x 7–22**	**middle**	**bending, twisting, pendulum-like**	**30–34**	**+**	**rounded**	**+**	**this study**
*S. terebellae*	*Terebella lapidaris*	E. Pacific, E. Atlantic	Intestine	spindle-shaped	30–330 x 12–40	oval (ellipsoidal)	35 x 15	posterior half	Bending, twisting	46.00	+	Pointed to slightly round	+	[3, 26]
*S. hollandei*	*Sabellaria alveolata*	E. Atlantic	Intestine	spindle-shaped, elongate, flattened	up to 500 x 20–30	spherical or ovoid	16 x 6–8	?	bending, twisting	about 16	?	heart-shaped	-	[3]
***S. hollandei***	***Sabellaria alveolata***	**E. Atlantic**	**Intestine**	**elongated, flattened**	**97–411 x** **13–43**	**spherical or ovoid**	**4–19 x** **6–17**	**middle to posterior half**	**bending, twisting, rapid rolling**	**18**	**+**	**heart-shaped, sometimes rounded**	**+**	**this study**
*S. sabellariae*	*Sabellaria alveolata*	E. Atlantic	Intestine	spindle-shaped	220–500 x 16–25	ovoid	16 x 13	?	bending, twisting	?	+	pointed, spine present	-	[3]
***S. sabellariae***	***Sabellaria alveolata***	**E. Atlantic**	**Intestine**	**spindle shaped, slightly flattened**	**68–227 x** **8–23**	**ovoid**	**7–15 x** **6–13**	**middle**	**quick coiling & uncoiling**	**14-Dec**	**+**	**pointed**	**+**	**this study**
*S. neosabellaria*	*Neosabellaria cementarium*	W. Pacific	Intestine	vermiform	125–350 x 9–12	spherical	10	middle	bending, twisting, undulated	12-Oct	+	nipple-shaped	+	[49]
*S. sabellae*	*Sabella pavonina*	E. Atlantic	Intestine		24–120 x 8–60	spherical	?	?	?	24–40	?	conical	-	[3]
***S. sabellae***	***Sabella pavonina***	**E. Atlantic**	**Intestine**	**rectangualar', flattened**	**40–85 x** **10–16**	**oval**	**6–12 x** **5–11**	**middle**	**bending, contracting**	**20–40**	**-**	**cone-shaped**	**+**	**this study**
*S. fallax*	*Cirriformia tentaculata*	E. Atlantic	Intestine	spindle-shaped	300–500 x 10–30	?	?	?	bending, twisting	90	?	knob-like	-	[3,26]
***S. fallax***	***Cirriformia tentaculata***	**E. Atlantic**	**Intestine**	**spindle-shaped**	**116–264 x** **10–26**	**ellipsoid**	**3–9 x** **6–17**	**anterior third**	**twisting. Quick coiling**	**40–45**	**-**	**flat-topped tip**	**+**	**this study**
*S. idanthyrsae*	*Idanthyrsus alveolata*	W. Pacific	Intestine	spindle-shaped, partially flattened	450–543 x 9–11	spherical	13–16 x 9–10	middle to posterior half	bending, twisting	20–22	+	pointed	+	[26]
*S. melongena*	*Thelepus japonicus*	W. Pacific	Coelom	oval, 'eggplant-shaped'	30–155 x 10–41	spherical	19-Jul	middle	none	30	-	neck-like, pointed	+	[50]
*S. cf. mesnili*	*Myxicola infundibulum*	W. Pacific	Intestine	spindle-shaped	85–157 x 18–24	ellipsoidal	7–9 x 10–11	anterior to posterior	bending, coiling	22–24	+	pointed to round	+	[26]
*S. boccardiellae*	*Boccardiella ligerica*	W. Pacific	Intestine	spindle-shaped, partially flattened	87–250 x 10–12	ellipsoidal	10–12 x 4–6	anterior half	bending, coiling, thrashing	12-Oct	-	pointed	+	[26]
*S. sensimae*	*Spirobranchus giganteus*	E. Pacific	Intestine	spindle shaped	130–170 x 10–13	ellipsoidal	10 x 4–6	middle	slow bending, twisting	16–18	-	rounded to blunt	+	[49]
*S. cf.echinatum*	*Dodecaceria concharum*	W. Pacific	Intestine	spindle-shaped	95–205 x 8–11	spherical	10	anterior half	bending, twisting	12-Oct	-	nipple-shaped	+	[49]
***S. spiralis sp. n.***	***Amphitritides gracilis***	**E. Atlantic**	**Intestine**	**spindle-shaped**	**105–194 x** **21–37**	**ovoid**	**10–21 x 15–25**	**posterior half**	**little bending**	**6 (12)**	**+**	**rounded**	**+**	**this study**
***S. antevariabilis sp. n.***	***Amphitritides gracilis***	**E. Atlantic**	**Intestine**	**spindle-shaped**	**131–190 x** **22–31**	**ovoid**	**13–21 x 15–20**	**middle**	**little bending, plastic mucron**	**6**	**?**	**plastic**	**+**	**this study**
***S. opheliae sp. n.***	***Ophelia roscoffensis***	**E. Atlantic**	**Intestine**	**sugar snap pea-like'**	**123–177 x** **10–16**	**ovoid**	**10–16 x** **7–11**	**middle to posterior half**	**little bending**	**none**	**+**	**elongated, rounded**	**+**	**this study**

**Fig 2:**
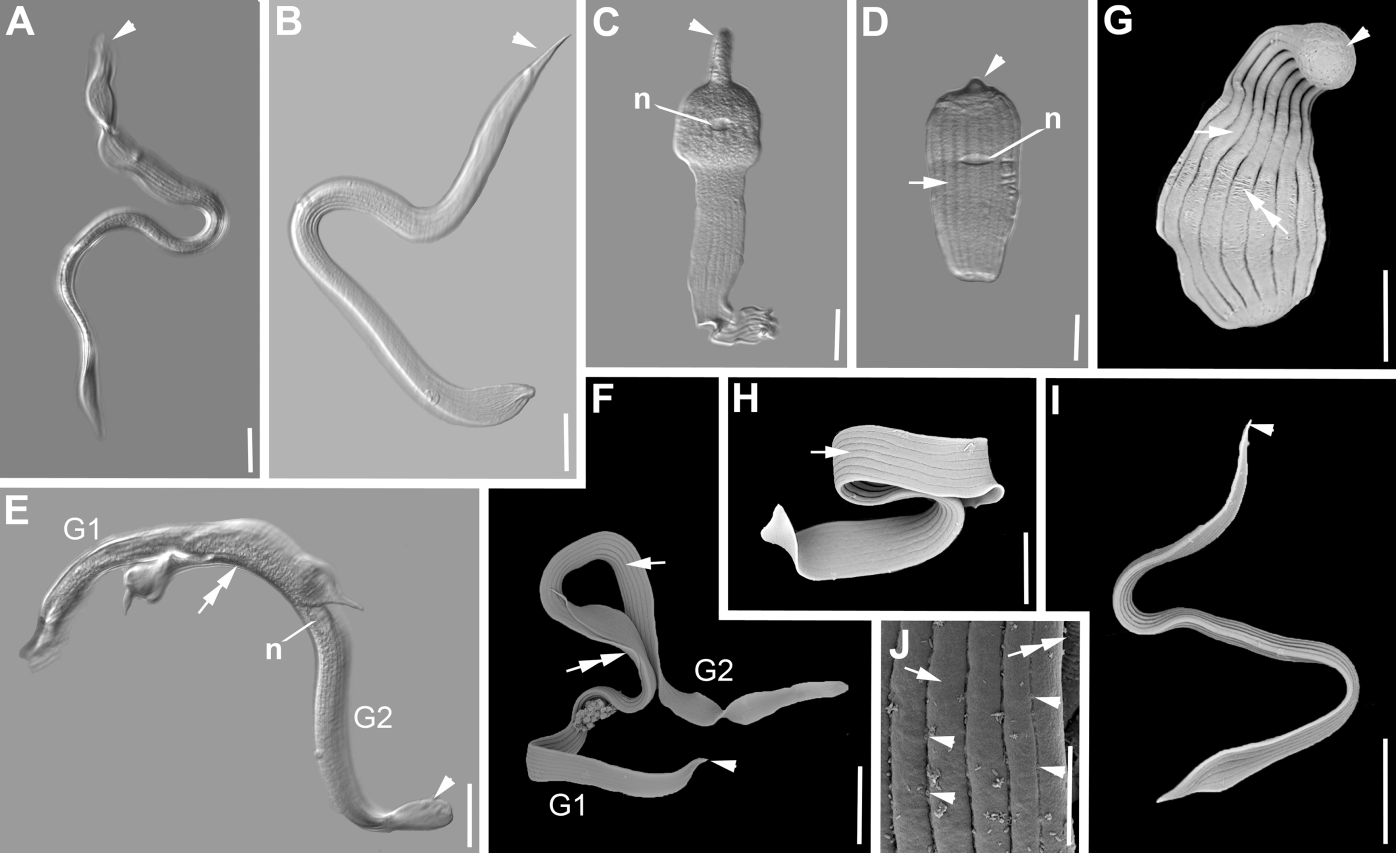
Differential interference contrast (DIC) light micrographs and scanning electron micrographs (SEM) showing the general morphology and surface ultrastructure of the gregarine *Selenidium hollandei* isolated from the polychaete *Sabellaria alveolata*. **A-B.** DIC micrographs of elongated and flattened trophozoites. The anterior end (mucron area, arrowhead) is narrower than the posterior end of the cell. **C-D.** Contracted trophozoites during peristaltic movement. The ovoid nucleus is visible (n). Broad epicytic folds (arrow) inscribe the surface of the entire cell. **E-F.** DIC and SEM micrographs of two gamonts (G1, G2) in lateral syzygy with overlapping posterior ends (double arrow). **G-I.** Trophozoites in different stages of movement. When contracted or bended, transverse striations (double arrow) form on the surface. The mucron (arrowhead) at the anterior end is free of folds. The broad longitudinal epicytic folds (arrows) cover most of the body. The lateral view shows dorso-ventrally extremely flattened trophozoites. **J.** Longitudinal epicytic folds (arrow) with visible pores (arrowheads) in the grooves between the folds. Scale bars: Fig 2A, C-D, G-H, 20 μm; Fig 2B, E-F, 30 μm; Fig 2I, 25 μm; Fig 2J, 5 μm.

***Selenidium hollandei*** (Fig 2, Table 1). The trophozoites of this archigregarine were isolated from the intestines of the polychaete *Sabellaria alveolata* (Linnaeus, 1767). The morphology matched the original description of *S*. *hollandei* by Vivier and Schrével [13] and several accounts in studies on different aspects that followed [3,10,11]. Trophozoites were mostly elongated and extremely flattened (Fig 2A, 2B, 2H and 2I). They were 250 μm (97–411 μm, n = 25) long and 22 μm (13–43 μm, n = 25) wide (Fig 2A–2D). The anterior and posterior end were both heart-shaped, but the anterior end was often a lot narrower than the posterior end (Fig 2B and 2I). The anterior end showed a slight middle ridge in some specimens (Fig 2). In some cases the anterior end was rounded and swollen and the posterior end was blunt (Fig 2D). The nucleus was spherical or ovoid and measured 9 x 10 μm (4–19 x 6–17 μm, n = 6) (Fig 2C–2E), situated in the middle of the trophozoite or slightly shifted towards the posterior end. The SEM showed broad epicytic folds covering the cell (Fig 2F–2I). The density of the folds was up to 1 fold/micron (Fig 2H and 2J). None of the folds terminated on either end of the cell. When the trophozoites were maximally contracted there were densely packed transverse striations/folds visible (Fig 2F). Micropores were situated in the shallow grooves between the epicytic folds (Fig 2J). Syzygy between two cells was observed showing a dorso-ventrally overlap of the two posterior ends (Fig 2E and 2G). Single trophozoites showed rapid rolling movements.

**Fig 3:**
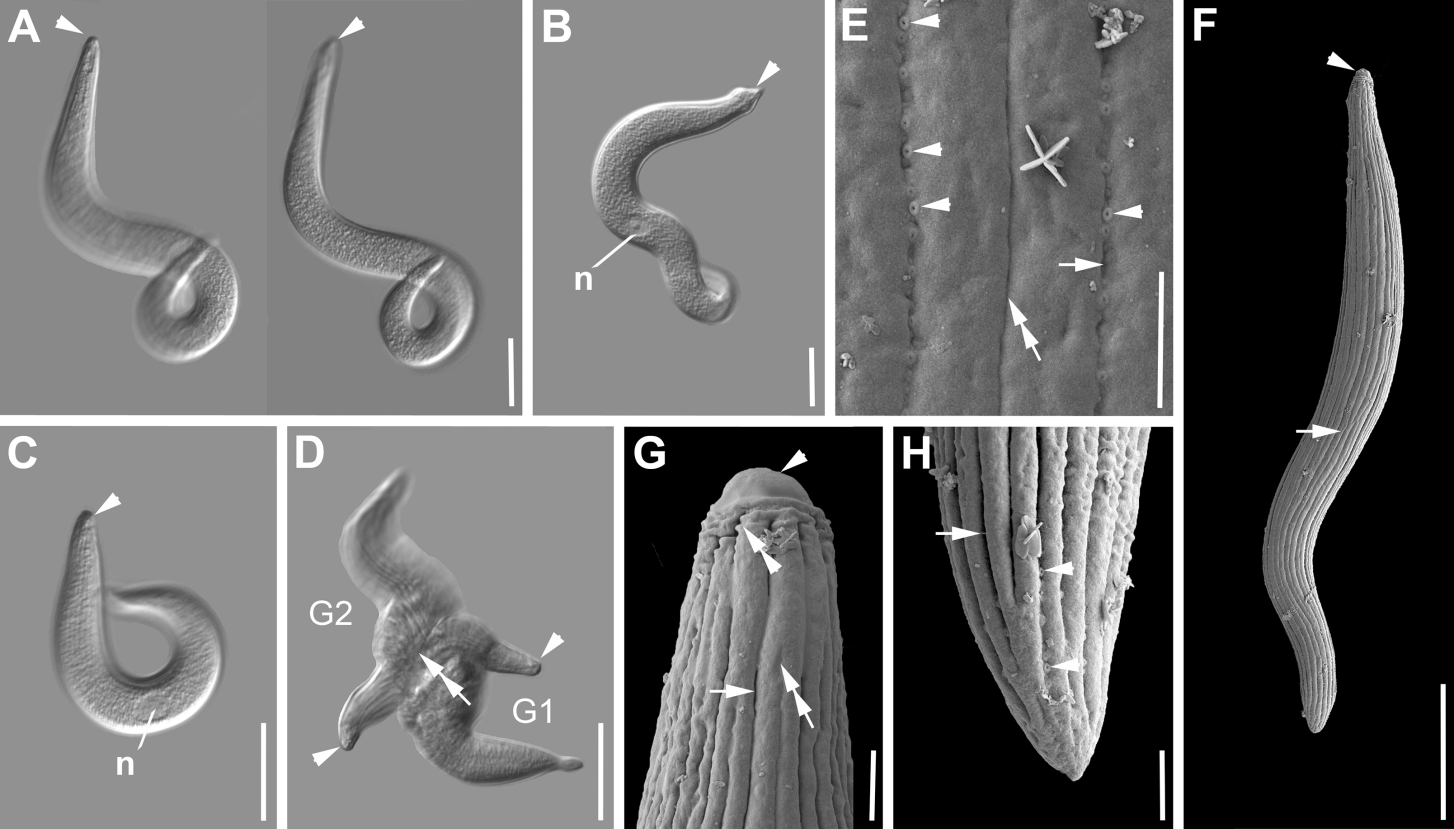
Differential interference contrast (DIC) light micrographs and scanning electron micrographs (SEM) showing the general morphology and surface ultrastructure of the gregarine *Selenidium sabellariae* isolated from the polychaete *Sabellaria alveolata*. **A- C.** DIC micrographs of trophozoites in different focal planes and different stages of movement. The mucron (arrowhead) is pointed to rounded and the ovoid nucleus (n) is situated in the middle of the cell. **D.** Two gamonts (G1, G2) in lateral syzygy with their orientation in opposite directions. The anterior ends are marked with arrowheads. Both gamonts are widest in the area of the junction (double arrow). **E.** Higher magnification SEM of the surface ultrastructure. There are numerous micropores (arrowheads) along the grooves of the main folds (arrow), whereas the grooves of the secondary folds (double arrow) do not display any micropores. **F.** Trophozoite, showing a rounded mucron (arrowhead) and longitudinal epicytic folds (arrow) with secondary folds expanding over parts of the cell’s length. **G.** Anterior end showing the rounded mucron (arrowhead) that is free of folds, but has a basal cluster of transverse striations (double arrowhead). Shortly after the mucron the main folds (arrow) start splitting (double arrowhead) into secondary folds. **H.** Posterior end of the trophozoite, showing the merging secondary folds (arrow), before they reach the posterior end. Scale bars: Fig 3A-D, 30 μm; Fig 3F, 20 μm; Fig 3E, G-H, 2 μm.

***Selenidium sabellariae*** (Fig 3, Table 1). Trophozoites of this archigregarine species were also isolated from the polychaete *Sabellaria alveolata*. The morphology matched the original description of *S*. *sabellariae* by Schrével [43] and accounts in later studies (e.g. [3]). The trophozoites were spindle-shaped and slightly flattened dorso-ventrally (Fig 3A–3C and 3F). Trophozoites were 137 μm (68–227 μm, n = 19) long and 16 μm (8–23 μm, n = 19) wide (Fig 3A and 3B). The anterior and posterior end both tapered into pointed tips. The ovoid nucleus 11 x 9 μm (7–15 x 6–13 μm, n = 15) situated in the middle of the cell contained a circular nucleolus [5 (4–6) μm in diameter, n = 10) (Fig 3B and 3C). The SEM revealed around 14 epicytic longitudinal folds (Fig 3F–3H). These folds split ‘superficially’ into two folds shortly after the mucron with rounded tip, which was free of folds, and merged again shortly before the posterior end (Fig 3G and 3H). There were numerous micropores visible in the grooves of the main folds, and none in the “superficially’ or secondary ones (Fig 3E). The density of the folds was 1 fold/micron at the anterior end (Fig 3G and 3H). Several transverse folds formed the base of the mucron (Fig 3G). Two cells in syzygy were laterally attached to each other with the gamonts oriented in opposite directions (Fig 3D). Single trophozoites showed quick coiling and uncoiling movements.

**Fig 4:**
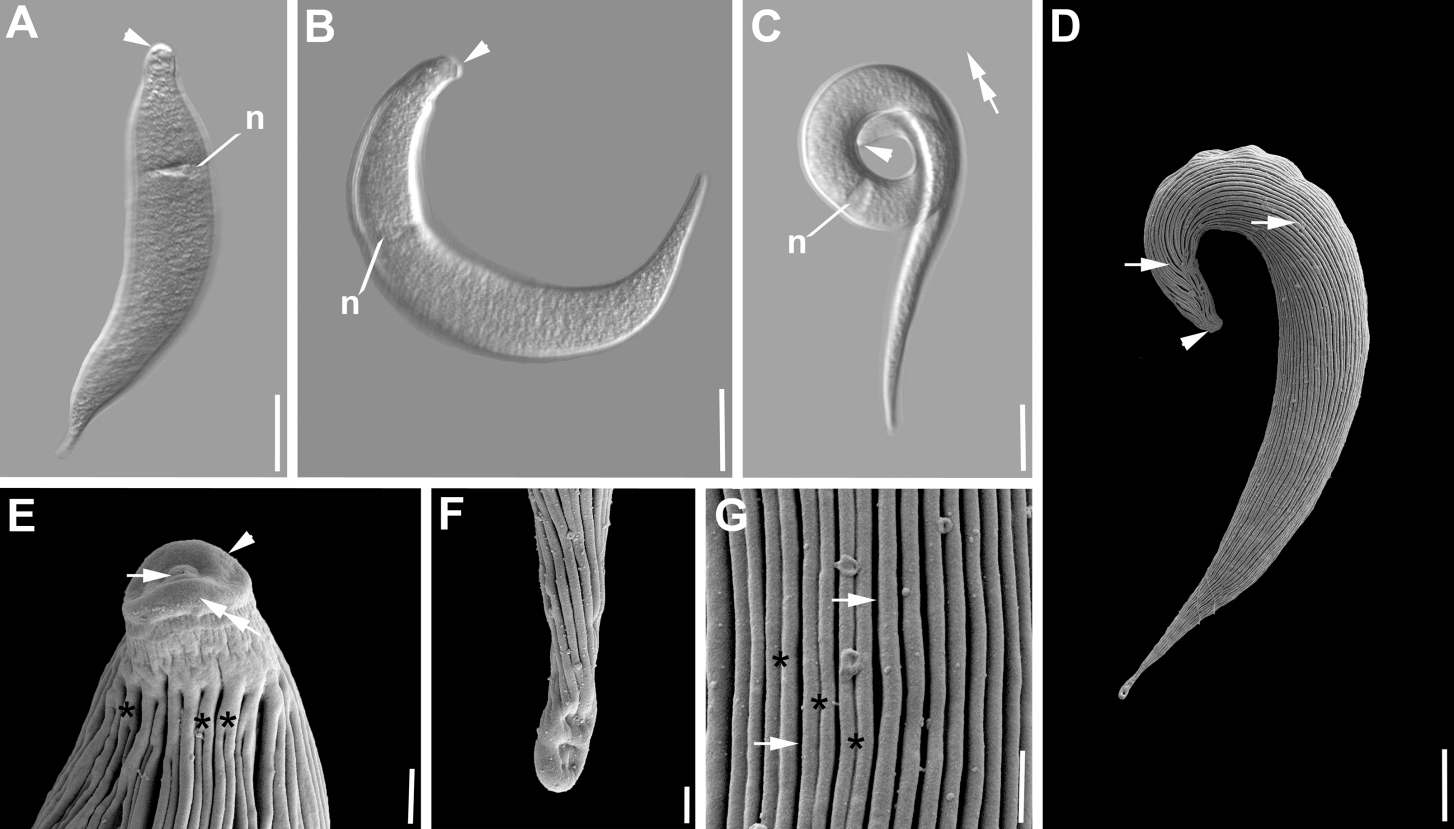
Differential interference contrast (DIC) light micrographs and scanning electron micrographs (SEM) showing the general morphology and surface ultrastructure of the gregarine *Selenidium fallax* isolated from the polychaete *Cirriformia tentaculata*. **A-C.** DIC micrographs of trophozoites in different stages of movement. Trophozoits can be elongated to curled. The mucron (arrowhead) is rounded and sometimes quite flattened at the tip. The ellipsoid nucleus (n) is situated in the anterior part of the cell. **D.** SEM micrograph of a trophozoite, showing a flattened mucron (arrowhead) and longitudinal epicytic folds (arrows) with secondary folds expanding over parts of the cell’s length. The posterior end is very pointed. **E.** Anterior end showing the flat-topped mucron (arrowhead) that is free of folds, but has a nipple-like structure (arrow) in the middle. The upper rim appears to be a bit bulgy (double arrowhead). The region directly after the bulge is inscribed by short, narrow, superficial folds. The broader main folds start splitting (asterisks) into secondary folds shortly after. **F.** Posterior end of the trophozoite showing the tip free of folds, but with some indentations. **G.** Higher magnification SEM of the surface ultrastructure. The grooves between the secondary folds (asterisks) are often narrower than the grooves between the main folds (arrows). Scale bars: Fig 4A-B, 25 μm; Fig 4C, 15 μm; Fig 4D, 10 μm; Fig 4E-G, 1 μm.

***Selenidium fallax*** (Fig 4, Table 1). Trophozoites of this archigregarine species infect the intestines of the polychaete *Cirriformia tentaculata* (Montagu, 1808). The morphology matched the original description of *S*. *fallax* by MacGregor & Thomasson [44] and accounts on different aspects that followed (e.g. [3,28]). The trophozoites were spindle-shaped (Fig 4A–4D). Trophozoites were 174 μm (116–264 μm, n = 23) long and 16 μm (10–26 μm, n = 23) wide (Fig 4A–4D). The anterior end tapered into a flat-topped tip with a nipple-like structure in the middle (Fig 4A, 4B and 4E). The posterior end tapered into a pointy tip (Fig 4A–4D and 4F). The ellipsoid nucleus 5 x 11 μm (3–9 x 6–17 μm, n = 21) situated in the anterior third of the cell (Fig 1A–1C) contained a circular nucleolus [5 (4–5) μm in diameter, n = 7]. The SEM revealed around 40 to 45 epicytic longitudinal folds (Fig 4D and 4G) and the mucron being free of folds (Fig 4E). The surface right beneath the bulging rim of the mucron was inscribed by short, narrow, superficial, longitudinal folds (4E). The main folds emerged just after this area, split subsequently into two folds and merged again shortly before the posterior end (4F). The main grooves also called ‘primary’ grooves were deeper and wider than the superficial ‘secondary’ grooves (Fig 4E and 4G). Micropores were only visible in the ‘primary grooves’. The density of the folds was up to 2 doublefolds/micron (Fig 4G). Free trophozoites showed quick coiling and uncoiling movements (Fig 4C). The motion also involved some helical twisting. The anterior end of the cell was always the center of the formed spiral (Fig 4C). After the trophozoites had been isolated for some time, they often remained in the coiled position.

**Fig 5:**
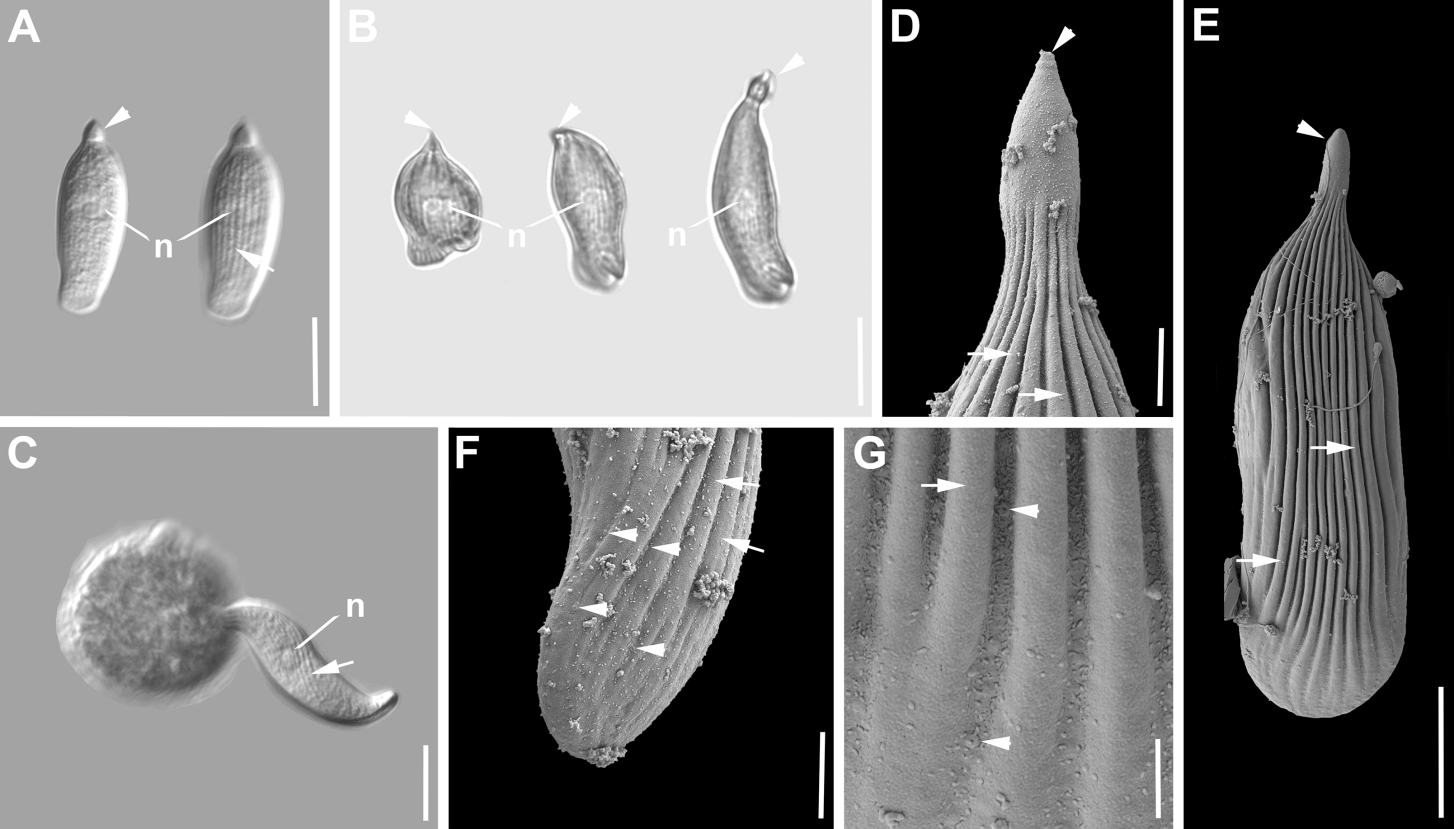
Phase contrast (PC), differential interference contrast (DIC) light micrographs and scanning electron micrographs (SEM) showing the general morphology and surface ultrastructure of the gregarine *Selenidium sabellae* isolated from the polychaete *Sabella pavonina*. **A.** DIC micrographs of trophozoites in different focal planes. The mucron (arrowhead) is cone-shaped. The ellipsoid nucleus (n) is situated in the middle of the cell. Longitudinal epicytic folds (arrow) are visible along the cell. The posterior part ends in a dorso-ventrally flattened, blunt end. **B.** PC micrographs showing the plasticity of the cell. The nucleus (n) keeps its position in the middle of the trophozoite. **C.** DIC micrograph of an attached trophozoite with visible epicytic folds (arrow). **D.** High magnification SEM of the anterior end showing the cone-shaped mucron (arrowhead) that is free of folds. The longitudinal epicytic folds (arrows) start to broaden after their emergence. **E.** Trophozoite, showing the cone-shaped mucron (arrowhead) and longitudinal epicytic folds (arrows) that fan out right after the mucron. The posterior end is broadly rounded or blunt. **F.** Posterior end of trophozoite in lateral view, showing that the very tip is free of folds. The cell is dorso-ventrally flattened. There are micropores (arrowheads) visible in between the folds. **G.** Surface ultrastructure towards the posterior end. The epicytic folds (arrow) enlarge towards the posterior end, and gradually terminate. There are numerous micropores (arrowheads) along the grooves between the folds. Scale bars: Fig 5A, C, 20 μm; Fig 5B, 30 μm; Fig 5E, 10 μm; Fig 5F, D, 3 μm; Fig 5G, 1 μm.

***Selenidium sabellae*** (Fig 5, Table 1). Trophozoites of this archigregarine species were isolated from the intestines of the polychaete *Sabella pavonina* Savigny, 1822. The morphology matched the original description of *S*. *sabellae* by Lankester [45], its re-description by Ray [46], and several accounts in studies that followed (e.g. [3]). The cells were short and slightly dorso-ventrally flattened (Fig 5A, 5E and 5F). Trophozoites were 61 μm (40–85 μm, n = 22) long and 14 μm (10–16 μm, n = 23) wide. The anterior end tapered into a narrow, but long, cone-shaped tip. The posterior end was broadly rounded or blunt (Fig 5A and 5D–5F). The ovoid nucleus 9 x 7 μm (6–12 x 5–11 μm, n = 21) situated in the middle of the cell (Fig 5A and 5B), contained a circular nucleolus [4 (3–6) μm in diameter, n = 18]. In some cases the trophozoites were attached to a globular body (Fig 5C). The SEM revealed around 20–40 epicytic longitudinal folds (Fig 5E). The folds were quite narrow starting at the base of the mucron (free of folds), and enlarged towards the posterior end, where they terminated gradually (Fig 5D–5F). There were numerous micropores scattered along the grooves of the epicytic folds (Fig 5F and 5G). The density of the folds was 1 fold/micron at the anterior end (Fig 5E). Syzygy between two cells was observed showing a dorso-ventrally overlap of the two posterior ends. Trophozoites were able to bend, as well as expand and contract along their length (Fig 5B).

**Fig 6:**
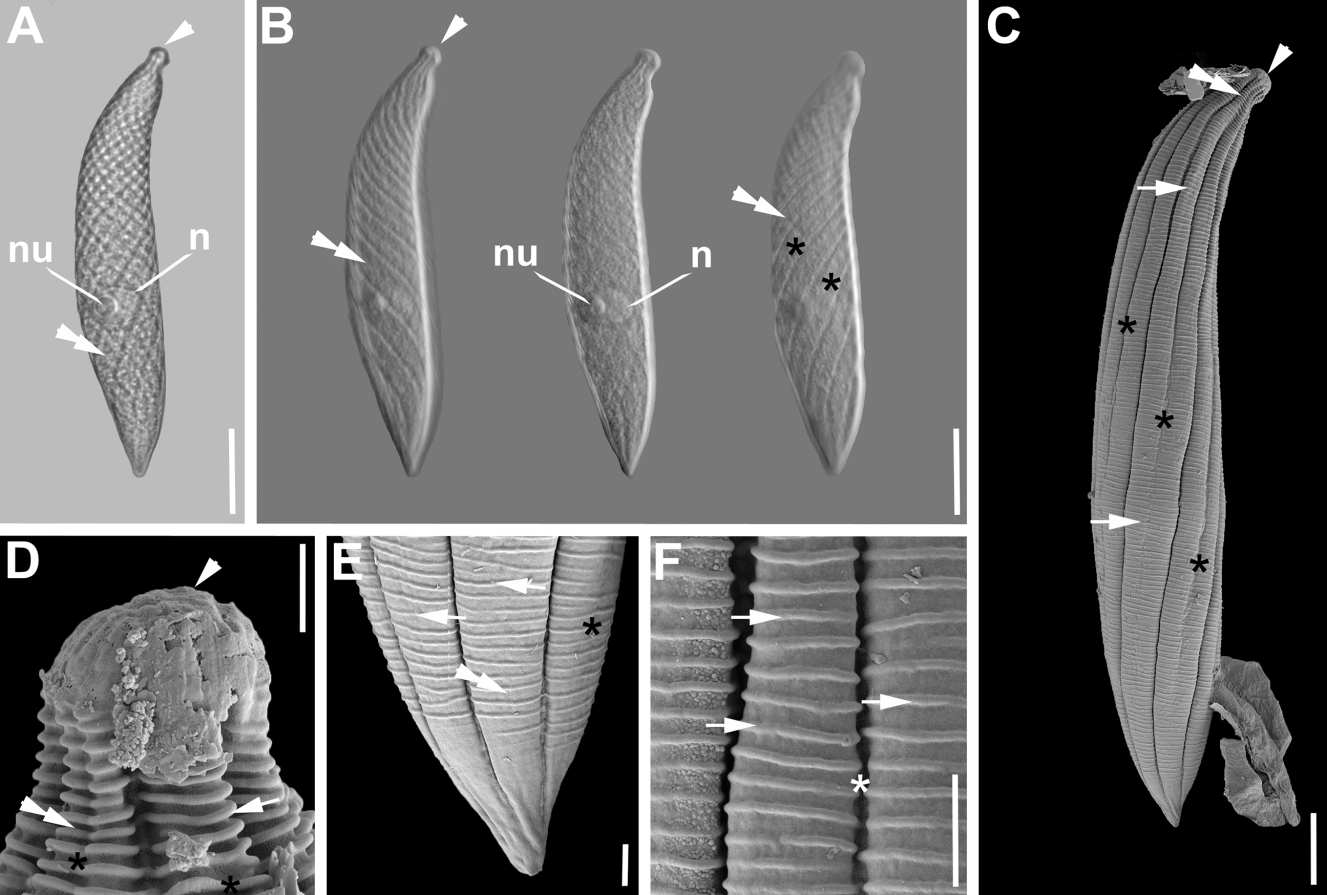
Phase contrast (PC), differential interference contrast (DIC) light micrographs and scanning electron micrographs (SEM) showing the general morphology and surface ultrastructure of the gregarine *Selenidium spiralis* sp. n. isolated from the polychaete *Amphitritides gracilis*. **A.** PC micrograph showing the general spindle-like shape of the trophozoites with spirally arranged epicytic folds (double arrowhead) that appear to overlap each other in a crisscross pattern. The ovoid nucleus (n) with its round nucleolus (nu) is situated in the posterior half of the cell. The mucron (arrowhead) is set apart from the rest of the cell through a slight indentation. **B.** DIC micrographs of the same cell as in A in different focal planes. The main epicytic folds (double arrowhead) and the secondary epicytic folds (asterisks) are spirally arranged along the longitudinal axis of the cell. **C.** SEM micrograph showing the surface ultrastructure of the trophozoite. The mucron (arrowhead) is free of folds. The main epicytic folds (double arrowheads) start right after the mucron and split very early into secondary folds. The grooves (asterisks) between the secondary folds appear quite deep from the anterior end and become gradually shallower towards the posterior end at which point they disappear completely. The cell is adorned with transverse striations (arrows) along its whole length apart from the anterior and posterior tip. **D.** The mucron (arrowhead) is free of folds. The very prominent transverse striations (arrow) start immediately posterior to the mucron, similar to the main epicytic folds (double arrowhead) and the split (asterisks) into secondary folds soon after. **E.** The transverse striations (arrows) continue to almost the tip of the posterior end, but are less prominent compared to the anterior end. One slight indentation (asterisk) of a secondary fold is visible on one of the main epicytic folds (double arrowhead). **F.** High magnification SEM of the transverse striations (arrow) and the grooves (asterisk) between the folds. Scale bars: Fig 6A-B, 25 μm; Fig 6C, 10 μm; Fig 6D-F, 2 μm.

***Selenidium spiralis* sp. n.** (Fig 6, Table 1). Trophozoites were isolated from the polychaete *Amphitritides gracilis* (Grube, 1860). The cells were spindle-shaped (Fig 6A–6C). Trophozoites were 139 μm (105–194 μm, n = 18) long and 31 μm (21–37 μm, n = 18) wide. The anterior end narrowed into a rounded mucron (Fig 6B and 6D) with a slight indentation setting it apart from the rest of the cell. The posterior end tapered into a more pointed tip (Fig 6E). The cells were wider in the posterior part of the trophozoite compared to the anterior part (Fig 6A–6C). The ovoid nucleus 12 x 20 μm (10–15 x 15–25 μm, n = 18) situated in the posterior half of the cell (Fig 6A and 6B), contained a circular nucleolus [7 (5–9) μm in diameter, n = 9). The trophozoites appeared to have a fishnet pattern on the surface in some of the micrographs (Fig 6A and 6B). The SEM revealed six epicytic longitudinal folds (Fig 6B and 6C). These folds split into two folds shortly after the mucron, which was free of folds, and merged again shortly before the posterior end (Fig 6A–6F). The main grooves also called ‘primary’ grooves were deeper than the superficial ‘secondary’ grooves (Fig 6C). The grooves between the secondary folds become gradually shallower towards the posterior end at which point they disappear completely (Fig 6C). The folds were more or less helically arranged along the cells body (Fig 6B and 6C). Micropores were observed in the ‘primary grooves’. The density of the folds was less than 1 fold/micron (Fig 6F). The epicytic folds were covered in thick, evenly spread transverse striations (2–3 transverse striations/micron), which started right after the mucron (Fig 6C–6F). The posterior end was free of the transverse striations. The striations became less prominent over the length of the cell (4D-F). Trophozoites were not very active and showed some bending movements.

**Fig 7:**
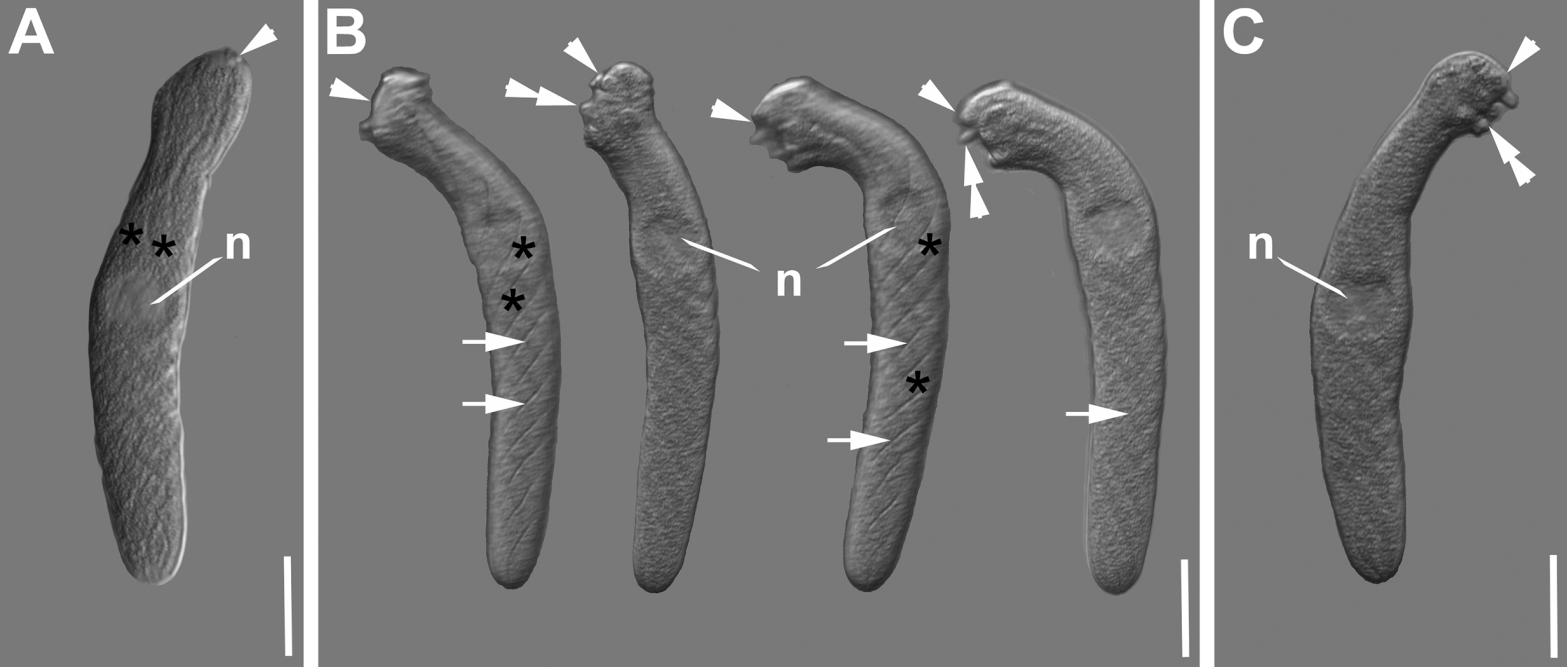
Differential interference contrast (DIC) light micrographs showing the general morphology of the gregarine *Selenidium antevariabilis* sp. n. isolated from the polychaete *Amphitritides gracilis*. **A-C.** DIC micrographs of spindle-shaped trophozoites with plastic anterior (arrowhead) and rounded posterior ends. The arrowhead marks the mucron area, which can be rounded or flattened, but often showing finger-like protrusions (double arrowhead). The ovoid nucleus (n) is situated in the middle of the cell or slightly shifted to the anterior end. Broad longitudinal epicytic folds (asterisks) run along the cells anterior-posterior axis with a helical turn, which appears like a criss-cross pattern on the surface (arrow). Scale bars: Fig 7A-C, 30 μm.

***Selenidium antevariabilis* sp. n.** (Fig 7, Table 1). Trophozoites were isolated from the polychaete *Amphitritides gracilis* (Grube, 1860). The cells were spindle-shaped, sometimes with a slight indentation at the anterior end superficially separating the mucron area from the rest of the cell (Fig 7A). Trophozoites were 165 μm (131–190 μm, n = 7) long and 29 μm (22–31 μm, n = 7) wide (at the widest part of the cell). The anterior end was very plastic in shape, changing from almost rounded to flat with finger-like protrusions, while the posterior end was rounded (Fig 7A–7C). The ovoid nucleus [17 x 19 μm (13–21 x 15–20 μm) in diameter, n = 7] was situated in the middle of the cell (Fig 7A–7C). The trophozoites were widest at the position of the nucleus. The nucleus contained a circular nucleolus [9 (7–11) μm in diameter, n = 4]. The trophozoites appeared to have a fishnet pattern on the surface in some of the micrographs (Fig 7A and 7B). Six epicytic longitudinal folds were more or less helically arranged along the cells body (Fig 7B and 7C). This helical arrangement of the folds leads to the visible fishnet pattern, due to a crisscross overlap of the longitudinal folds on opposite cell surfaces. The density of folds was less than 1 fold/micron (Fig 7B). Trophozoites showed some bending movements and very active change of the morphology of the anterior end (Fig 7A and 7C).

**Fig 8:**
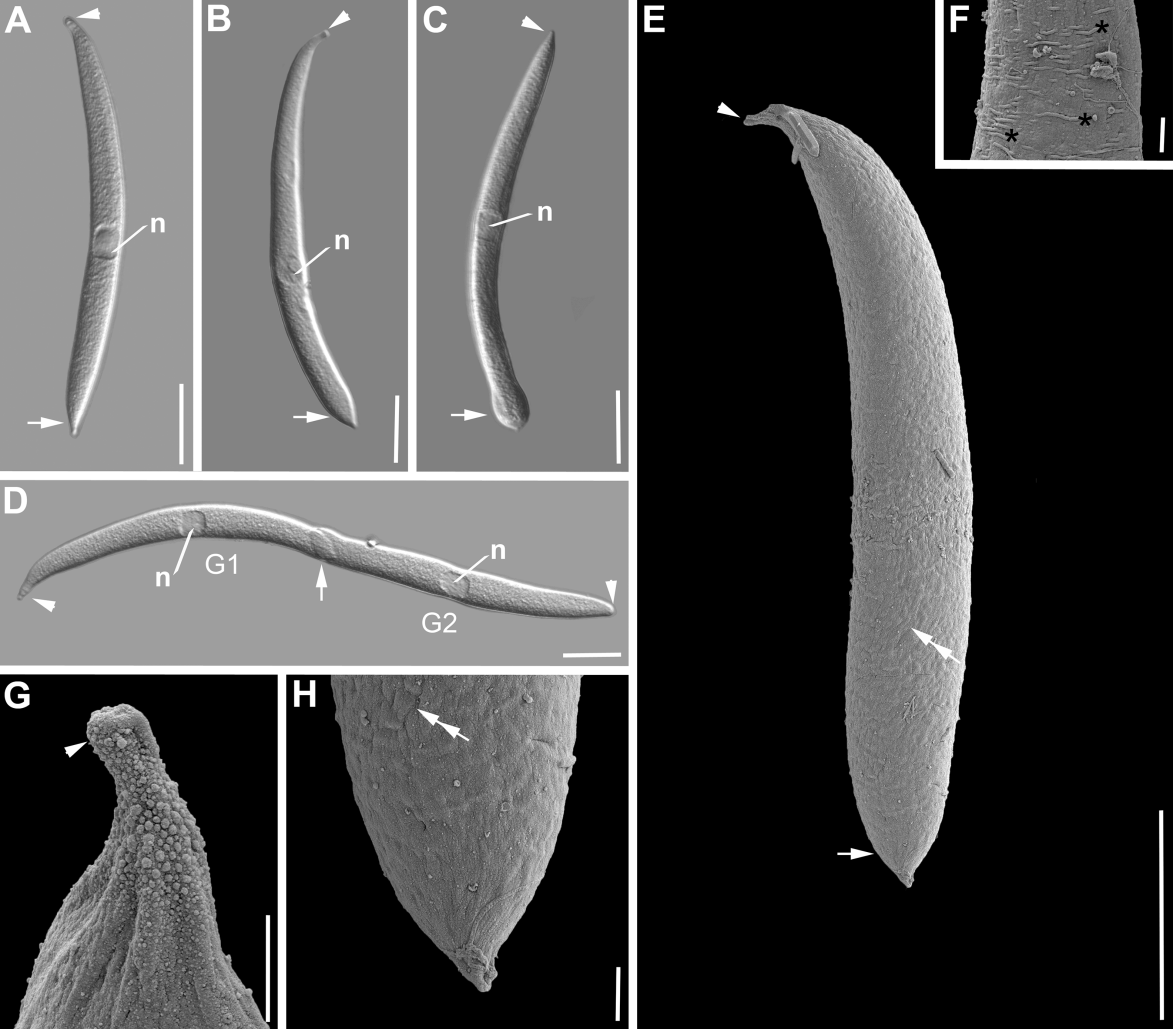
Differential interference contrast (DIC) light micrographs and scanning electron micrographs (SEM) showing the general morphology and surface ultrastructure of the gregarine *Selenidium opheliae* sp. n. isolated from the polychaete *Ophelia roscoffensis*. **A-C.** DIC micrographs of different trophozoite cells showing the general elongated cell shape that is slightly reminiscent of a pea pod. The mucron (arrowhead) anterior end is slightly elongated and ends in a rounded tip, while the posterior end (arrow) is wider and either blunt or heart-shaped. The ovoid nucleus (n) is situated in the middle of the cell or shifted slightly to the posterior end. **D.** Two gamonts (G1, G2) in syzygy. This species forms a caudo-lateral syzygy with the two posterior ends overlapping (arrow). Both gamonts have a visible nucleus (n) in the middle. **E.** SEM micrograph showing the general morphology and ultrastructure of the trophozoite, with an elongated mucron (arrowhead) and a heart- shaped (arrow) posterior end. The surface seemed to be crinkled in places (double arrow). **F.** Some surface areas showed few transverse striations (asterisks) but in no obvious pattern. **G.** Anterior end with elongated mucron (arrowhead). **H.** Posterior end with visible crinkles (double arrow) on the surface. Scale bars: Fig 3A–3D, 30 μm; Fig 3E, 20 μm; Fig 3F–3H, 2 μm.

***Selenidium opheliae* sp. n.** (Fig 8, Table 1). Trophozoites were isolated from the polychaete *Ophelia roscoffensis* Augener, 1910. The cell shape was generally elongated to slightly reminiscent of a pea pod (Fig 8A–8C and 8E). Trophozoites were 151 μm (123–177 μm, n = 19) long and 13 μm (10–16 μm, n = 19) wide. The anterior end tapered into an elongated, but at the tip mostly rounded mucron (Fig 8A–8C and 8G). The posterior end was wider with a heart-shaped or blunt end (Fig 8C, 8E and 8H). The ovoid nucleus 13 x 9 μm (10–16 x 7–11 μm, n = 19) situated in the middle of the cell or slightly shifted to the posterior end contained a circular nucleolus [6 (5–8) μm in diameter, n = 9] (Fig 8A–8D). The SEM revealed no epicytic folds (Fig 8F–8H). The surface of some trophozoites appeared a bit crinkled (Fig 8E and 8H). On some of the trophozoites transverse striations were visible (Fig 8F). Syzygy of two cells was observed, in which the outer posterior ends were attached through overlap (Fig 8D). Trophozoites were not very active and showed some bending movements.

### Molecular phylogenetic analyses

The preliminary phylogenetic analysis of the pan-apicomplexan dataset revealed an increased rate of evolution in gregarines compared to the already highly divergent crown apicomplexan lineages. The averaged root-to-tip distance of gregarines as a whole, or archigregarines, was significantly higher than in other lineages (S1 Fig). Together with the non-homogenous base composition observed in several gregarine species, this makes the correct phylogenetic reconstruction of apicomplexans very challenging. Therefore, together with the widely-used Maximum likelihood inference under the gamma-corrected GTR model, we have employed also the CAT admixture model (or rather the ‘C40’ approximation of CAT; see relevant Methods part for details and reasoning), which is supposed to be more robust to phylogenetic artifacts stemming from varying rates of evolution along the phylogenetic tree, namely long-branch attraction (LBA).

**Fig 9:**
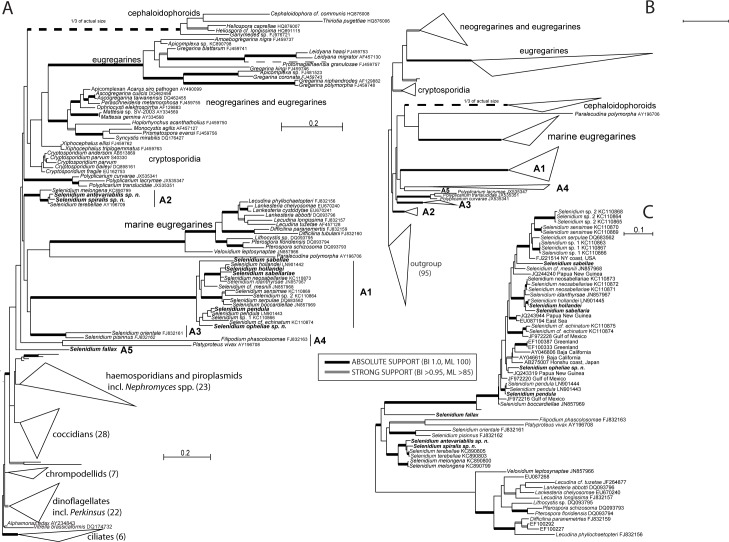
Phylogenetic relationships of apicomplexans. **A.** Bayesian tree of apicomplexans inferred using the GTR + C40 model of substitution in Phylobayes on an alignment of 171 small subunit (SSU) rDNA sequences and 1488 unambiguously aligned sites. Thickened lines at branches denote Bayesian posterior probabilities and bootstrap support (see legend in the center of the figure for details). The eight sequences derived from this study are highlighted in bold. A1 to A5 show the positions of the five ‘archigregarine’clades. **B.** Schematized Maximum likelihood phylogeny analysis of the same dataset inferred under the GTR+G model as implemented in RAxML 8.2. The outgroup composition and topology is the same as in Fig 9A and is simplified due to space limitations. **C.** Bayesian topology of the dataset **S** with exhaustive sampling of Selenidiidae, also enriched for environmental sequences inferred in Phylobayes under the GTR + C40 model of substitution. The branching support follows parameters of tree **A**.

The resulting phylogeny (Fig 9) shows the expected topology with monophyletic myzozoans (dinozoans, chrompodellids [47] and apicomplexans) and ciliates as an outgroup. Both dinozoans and apicomplexans are monophyletic, while chrompodellids split into two clades. One, represented by *Alphamonas* and *Vitrella*, which branch out as the basal-most myzozoan lineage, whereas the rest (*Chromera*, *Voromonas* and *Colpodella*) are a sister group to the apicomplexans *s*.*s*.

Apicomplexans are further split into two clades. The first contains ‘true’ coccidians, haemosporidians with *Nephromyces* spp. and the adeleorinid coccidians (*Adelina* and *Hepatozoon*). The second clade is comprised of gregarines including the cryptosporidians and two highly divergent apicomplexans *Platyproteum vivax* and *Filipodium phascolosomae*, which show typical archigregarine features, but were classified as an independent myzozoan lineages by a previous study of Cavalier-Smith [1].

Within the gregarine clade, we were able to identify the three main currently recognized lineages (Archigregarinorida, Eugregarinorida, Neogregarinorida), as well as *Cryptosporidium* as sister clade to the gregarines from terrestrial hosts. Bayesian inference (Fig 9A) split gregarines into two main clades. The first contains neogregarines, cryptosporidia, cephaloidophorids and terrestrial gregarines. The second comprised of archigregarines and marine eugregarines. Finally, *S*. *fallax*, whose phylogenetic position is for the first time presented here, as well as *Platyproteum vivax* and *Filipodium phascolosomae* were the most basal gregarines and sister to the two above mentioned clades.

Maximum likelihood analysis split gregarines according to the ecology: terrestrial clade containing cryptosporidia, neogregarines and terrestrial eugregarines and the marine clade comprised of cephaloidophorids branching with marine eugregarines and several archigregarine lineages (Fig 9B). Both phylogenetic methods unequivocally and consistently placed the newly obtained sequences of *Selenidium* in three separate clades (Fig 9, S2 Fig, S3 Fig). *Selenidium pendula* branched within the well supported ‘true’ *Selenidium* clade with *S*. *sabellariae*, *S*. *sabellae*, *S*. *hollandei*, *S*. *opheliae* and nine other representatives, while *S*. *pisinnus* formed a weakly supported clade with *S*. *orientale* basal to it. Another putatively archigregarine clade with sequences related to *S*. *terebellae* (including two of our new isolates from *Amphitritides gracilis*) is found elsewhere among the gregarines. *Selenidium fallax* probably represents an independent (archi-) gregarine lineage of uncertain position. However, analysis of auxiliary datasets R and G (S2 Fig, S3 Fig) shows the gregarine topology is unstable and highly dependent on the phylogenetic method and site selection. It is worth mentioning that the archigregarine monophyly was also rejected using the au-test. While all the respective clades are robustly supported in all analyses, their mutual relationships are unclear to say the least and the support for deeper branching is missing completely. A pairwise distance calculation based on the Kimura two-parameter model [48] of 1656 nt (with pairwise exclusion of the indels) resulted in sequence divergences between 2% and 35.8% between all *Selenidium* species. The sequence divergence ranges were 2–15.6% (A1); 5.8–10.5% (A2) and 15.3 (A3) within clades A1-A3 and 18.5–35.8% between species from the different clades (S1 Table). Since the sequence of the archigregarine type species *S*. *pendula* has been recently published [33], we have also attempted to explore the diversity and phylogenetic structure of the genus *Selenidium s*.*s*. Our exhaustive sampling revealed that almost half of the diversity is comprised of environmental sequences without any morphology and host-specificity record. *Selenidium pendula* is found near the base of the *Selenidium* clade, which lacks a strong phylogenetic structure. It is interesting that while most archigregarine species are described from temperate regions, many of these environmental sequences originate from tropical and subtropical waters clearly showing their cosmopolitan distribution and research bias.

### Formal taxonomic description

Phylum Apicomplexa Levine, 1970

Subphylum Sporozoa Leuckart, 1879

Class Gregarinea J.A.O. Bütschli, 1882, stat. nov. Grassé, 1953

Order: Archgregarinorida Grassé, 1953

Family Selenidiidae Brasil, 1907

Genus *Selenidium* Giard, 1884

***Selenidium spiralis*** Rueckert and Horák **sp. n.**

urn:lsid:zoobank.org:act:0687AD8C-E669-47B4-8EC2-9FC22A039B42

**Diagnosis.** Trophozoites are spindle-shaped and 105–194 μm long (mean length 147 μm) and 21–37 μm wide (mean width 30 μm). Cell tapers into a rounded mucron at the anterior and a pointed tip at the posterior end. The ovoid nucleus (13 x 20 μm) is situated in the posterior half of the trophozoite. Trophozoites are brownish in colour under the LM due to the accumulation of amylopectin granules within the cytoplasm. Six longitudinally and helically oriented broad epicytic folds are present on the cell surface, except the mucron and the posterior tip. They are separated by deep, primary grooves and also show shallow secondary grooves in between. Epicytic folds are covered by thick transverse striations. The trophozoites are capable of some bending.

**DNA sequence.** The SSU rDNA sequence; GenBank Accession No. MF882900.

**Type locality.** Rock assemblages close to Station Biologique Roscoff (48°43’44”N, 3°59’23”W), Roscoff, France.

**Type habitat.** Marine.

**Type host.** *Amphitritides gracilis* (Grube, 1860) (Annelida, Polychaeta, Terebellidae).

**Location in host.** Intestinal lumen.

**Hapantotype.** Trophozoites on gold sputter-coated SEM stubs have been deposited in the Beaty Biodiversity Museum (Marine Invertebrate Collection) at the University of British Columbia, Vancouver, Canada (collection number MI-PR135).

**Iconotype.** Fig 6B.

**Etymology.** The species name *spiralis* refers to the helically arranged longitudinal epicytic folds.

***Selenidium antevariabilis*** Rueckert and Horák **sp. n.**

urn:lsid:zoobank.org:act:F830245F-4CF9-4925-9F20-CFFB8699DBCB

**Diagnosis.** Trophozoites are spindle-shaped and 131–190 μm long (mean length 165 μm) and 22–31 μm wide (mean width 29 μm). Cell tapers into a rounded posterior end. The anterior end is plastic in shape, being rounded, or flat, or showing finger-like protrusions. The ovoid nucleus (17 x 19 μm) is situated in the middle of the trophozoite and contains a circular nucleolus (9 μm in diameter). Trophozoites are brownish in colour under the LM due to the accumulation of amylopectin granules within the cytoplasm. Six longitudinally and helically oriented broad epicytic folds are present on the cell surface, except the mucron and the posterior tip. The trophozoites are capable of some bending and very active change of the morphology of the anterior end.

**DNA sequence.** The SSU rDNA sequence; GenBank Accession No. MF882905.

**Type locality.** Rock assemblages close to Station Biologique Roscoff (48°43’44”N, 3°59’23”W), Roscoff, France.

**Type habitat.** Marine.

**Type host.** *Amphitritides gracilis* (Grube, 1860) (Annelida, Polychaeta, Terebellidae).

**Location in host.** Intestinal lumen.

**Holotype.** The name-bearing type of this species is the specimen shown in Fig 7B (see Iconotype). This is in accordance with Declaration 45 recommendations to article 73 of the ICZN. An explanation is provided in the remarks.

**Iconotype.** Fig 7.

**Etymology.** The species epithet *antevariabilis* refers to the plastic morphology of the anterior end and stems from the Latin words ‘ante’ meaning front/before and ‘variabilis’ meaning changeable.

**Remarks.** In accordance with Declaration 45 of the ICZN we use the specimen presented in the light micrographs (7B) as name-bearing type material (Holotype). Here we provide reasoning, why no preserved specimen was used as name-bearing type for the new species. This new gregarine species occurred in very low numbers. The few specimens we were able to isolate were used for DNA extraction, prepared for SEM and for LM analyses. Trophozoites fixed for SEM often do represent a gregarine’s morphology better than a fixed and flattened cell on an object slide. Light micrographs (Iconotype) were obtained from freshly isolated cells and represent the morphology of the trophozoite and especially of the plasticity of the anterior end better than a fixed specimen on an object slide could. We were not able to find any gregarines on the filter prepared for SEM, therefore the only documentation of this species are the light micrographs and the cell measurements. We do provide the SSU rDNA sequence, which separates *S*. *antevariabilis* n. sp. clearly from *S*. *spiralis* n. sp. isolated from the same host. Therefore, we feel that we provide enough evidence for the description of a new species.

***Selenidium opheliae*** Rueckert and Horák **sp. n.**

urn:lsid:zoobank.org:act:8316105E-9419-4F06-9463-E12553F9E2C5

**Diagnosis.** Trophozoites elongated ellipsoid to pea pod shape and 123–177 μm long (mean length 151 μm) and 10–16 μm wide (mean width 13 μm). Anterior end is elongated with rounded mucron, posterior end is wide with heart-shaped or blunt end. The ovoid nucleus measures13 x 9 μm (10–16 x 7–11 μm, n = 19) and is situated in the middle of the cell or slightly shifted to the posterior end. Trophozoites are brownish in colour reflecting an accumulation of amylopectin granules within the cytoplasm. Epicytic folds are lacking. Surface of trophozoites can appear crinkled and some show transverse striations. Trophozoites are capable of bending movements.

**DNA sequence.** The SSU rDNA sequence; GenBank Accession No. MF882904.

**Type locality.** Le Guillec (48°68´56´´N, 4°06´78´´W), France.

**Type habitat.** Marine.

**Type host.** *Ophelia roscoffensis* Augener, 1910 (Annelida, Polychaeta, Opheliidae).

**Location in host.** Intestinal lumen.

**Hapantotype.** Trophozoites on gold sputter-coated SEM stubs have been deposited in the Beaty Biodiversity Museum (Marine Invertebrate Collection) at the University of British Columbia, Vancouver, Canada (collection number MI-PR136).

**Iconotype.** Fig 8B.

**Etymology.** The species name *opheliae* refers to the genus of the polychaete type host *Ophelia roscoffensis* Augener, 1910.

## Discussion

In the last decade more and more information has been published about the archigregarines, which occur exclusively in marine habitats and are most likely the stem group from which all gregarine and maybe even all apicomplexans have derived [24–26,29,32,49,50]). Most of these publications synergize data on morphology, ultrastructure and SSU rDNA sequences to newly describe or re-describe species in the most comprehensive way possible to date. Almost all of the work has been done on the trophozoite stages, the most prominent and abundant life cycle stages. Recently work on other life-cycle stages of *S*. *pendula* has been published [33]. Molecular data of archigregarines are still very limited compared to other taxa.

### Justification for newly erected species

Trophozoites of *Selenidium spiralis* sp. n. and *S*. *antevariabilis* sp. n. were isolated from *Amphitritides gracilis*. No gregarines have so far been reported for this terebellid polychaete. The analyses of the SSU rDNA sequences available for gregarines of the genus *Selenidium* placed *S*. *spiralis* sp. n. and *S*. *antevariabilis* sp. n. into a clade with other *Selenidium* species from terebellid worms (*Thelepus japonicus*), namely *Selenidium terebellae* and *Selenidium melongena*. While *S*. *terebellae* is a typical archigregarine of the genus *Selenidium*, with few longitudinal epicytic folds, bending/twisting and coiling movements and transverse striations, *S*. *melongena* is quite different (see Table 1 and Wakeman et al. 2014). One of the most distinct features is that the 30–40 epicytic folds are helically arranged along the longitudinal axis of the cell and that no movement could be observed. The new species described here present different degrees of helically arranged epicytic folds, but a lot less in numbers (six broad folds, in one case with secondary grooves). Along the whole length of the epicytic folds of *S*. *spiralis* sp. n. there are thick transverse folds at regular intervals. While *S*. *spiralis* sp. n. and *S*. *antevariabilis* sp. n. were found within the intestine, *S*. *melongena* was predominantly found attached to the outer wall of the intestine [50]. The calculated sequence divergences ranged between 8.7–10.5% for *S*. *spiralis* sp n., *S*. *terebellae* and *S*. *antevirabilis* sp. n. compared to *S*. *melongena* (compare S1 Table). The lowest sequence divergence (2%) was actually found between two established species *S*. *idanthyrsae* and *S*. *neosabellariae*. Apart from their sequence divergence (6.8%), the biggest difference between the two new species is the morphology of the anterior end. While *S*. *spiralis* sp. n. has a rounded mucron, the anterior end of *S*. *antevariabilis* sp. n. is very plastic and changing in form constantly. Combining all evidence we are convinced that both isolated trophozoites represent new *Selenidium* species.

*Ophelia roscoffensis* has never been described as hosting any gregarine species. There are two *Rhytidocystis* species (Agamococcidiorida) described from opheliid worms, namely *R*. *opheliae* from *O*. *bicornis* [51] and *R*. *henneguyi* from *O*. *neglecta* [52], but their phylogenetic position is not quite certain yet [9]. Rotari et al. [53] described metchnikovellids (hyperparasites of gregarines) from a *Selenidium* species infecting *Ophelia limacina*, but the gregarine species was not formally described and only a line drawing is available. Compared to the other known *Selenidium* species our newly described *S*. *opheliae* sp. n. from *O*. *roscoffensis* has very different morphological features, as this is the only species that does not show any longitudinal epicytic folds (compare Table 1). The crinkled surface and the few transverse striations could be the result of the SEM fixation process. The sequence divergence showed values between 6.9% and 15.4% when comparing *S*. *opheliae* sp. n. with all other *Selenidium* species in clade A1. The distances between all *Selenidium* species in clade A1 ranged from 2% to 15.6% (S1 Table). The phylogenetic tree based on gregarine SSU rDNA sequences alone (Fig 9) shows clearly that this species has a distinct position from the other *Selenidium* species within the *S*. *pendula* clade of true *Selenidium* species, therefore validating the erection of the new species.

### Archigregarine phylogeny

The so called archigregarines (i.e. apicomplexans mostly with vermiform or spindle-shaped actively moving trophozoits), represented here by all species with available SSU rDNA sequences, apparently comprise of five independent lineages: 1) a clade around the *Selenidium* type-species *S*. *pendula*, 2) a clade containing *S*. *pisinnus* and *S*. *orientale*, 3) a clade with parasites isolated from polychaetes *Amphitritides gracilis* and *Thelepus japonicus* and 4) a clade made up of two very divergent species *Platyproteum vivax* and *Filipodium phascolosomae*, and 5) the single sequence of *S*. *fallax* at the base of all other gregarines. Although all these lineages share similar morphology (see Table 1) and possibly (according to the limited insight available) also general biology, phylogenetic analysis of SSU rDNA clearly reveals well defined and separated lineages. However, the deeper branching reflecting their mutual relationship is again very unstable and poorly supported. All recent studies [1,25,26,33,49,50], as well as the phylogeny of our main dataset presented here (Fig 9) suggest their paraphyly. Archigregarine monophyly was also rejected by the approximately-unbiased test. However, short internal branches suggest rapid archigregarine diversification, which is always hard to capture using phylogenetic reconstruction. In our opinion, while improbable, the archigregarine monophyly cannot be reliably rejected based on current taxon sampling and SSU rDNA. One obvious outcome is that only the archigregarines of the clade around *S*. *pendula* should retain the genus name *Selenidium* and thorough revision of the whole archigregarine concept is required.

### True Selenidiidae vs. Selenidioides

Levine [3] split the genus *Selenidium* and its species into two: 1) *Selenidioides* within the family Selenidioididae in the order Archigregarinorida encompassing species with merogony and 2) *Selenidium* within the family Selenidiidae in the order Eugregarinorida without merogony. There have been many discussions before this split and ever since the split [16,25,26,29,30,32] about the taxonomy of gregarine species described as *Selenidium*, as it is difficult to prove or disprove the existence of merogony within a gregarine life-cycle. No sequence data were available back then and still the available DNA sequences are quite limited. Our phylogenetic trees based on SSU rDNA sequence data (Fig 9) show that *S*. *hollandei*, which after Levine [3] should belong to the genus *Selenidioides*, clusters within the big clade around the type species of the genus *Selenidium*, *S*. *pendula*, which was moved to the Eugregarinorida due to the ‘absence’ of merogony. Therefore, *S*. *pendula* as well as *S*. *hollandei* belong to the true Selendiidae, which has also been shown by Schrével et al. [33], inferring that the split of the genus *Selenidium* was premature, because it is not backed up by molecular sequence data and should therefore be ignored from hereon. While *S*. *sabellariae* and *S*. *sabellae* also cluster within the clade A1 together with *S*. *pendula*, the species *S*. *fallax* that should belong into this genus as well clusters out with any of the other ‘*Selenidium*’ sequences, which supports our suggestion to revise this genus at some point, when better/more sequence data and morphological descriptions of other species within this genus become available.

### Suitability of SSU rDNA for gregarine phylogeny

Molecular diversity is almost exclusively represented by SSU rDNA sequences for gregarine apicomplexans. Yet, even the most elaborate phylogenetic models designed for phylogenies of highly divergent datasets [54,55], failed to cope with the extreme diversity of gregarine SSU rDNA. The topology is unstable and is highly dependent on the particular model used (GTR + C40 vs. GTR) as well as taxon and site sampling. Therefore, we intentionally refrain from any taxonomic revisions of gregarines *s*.*l*. as recently proposed by Cavalier-Smith [1], as we do not find it justified by the available data. It is our opinion that we have to leave the question of relationships among the key gregarine lineages open until new sets of markers of sufficient sampling will be available.

## Supporting information

S1 Fig Boxplots showing averaged root-to-tip distances of main clades used in phylogenetic analyses. Three groups of gregarines (representatives of *Selenidium* s.s., archigregarines and gregarines as a whole) show significantly longer branch lengths and therefore also increased rates of evolution as revealed by pairwise t-test comparison to other clades presented in the dataset.
(EPS)

S2 Fig Phylogenetic topology of auxiliary dataset G (945 sites) as revealed by Bayesian inference under the GTR + C40 model as implemented in Phylobayes. Thickened lines show branching supported by Bayesian posterior probabilities (dark, p.p. 1.0, grey p.p. above 0.94). See relevant parts of methods for details.
(EPS)

S3 Fig Phylogenetic topology of auxiliary dataset R (1805 sites) as revealed by Bayesian inference under the GTR + C40 model as implemented in Phylobayes. Thickened lines show branching supported by Bayesian posterior probabilities (dark, p.p. 1.0, grey p.p. above 0.94). See relevant parts of methods for details.
(EPS)

S1 Table Estimates of evolutionary divergence between sequences. The numbers of base substitutions per site between sequences are shown. The analysis involved 50 nucleotide sequences. All ambiguous positions were removed for each sequence pair. There were a total of 1654 positions in the final dataset. The sequences for taxa in bold were derived from this study, taxa highlighted in light grey belong to *Selenidium* s.s. and taxa highlighted in dark grey are novel species for the first time described here.
(XLSX)
